# Influence of biopsychosocial factors on self-reported anxiety/depression symptoms among first-generation immigrant population in the U.S.

**DOI:** 10.1186/s12889-024-18336-w

**Published:** 2024-03-15

**Authors:** David Adzrago, Kiran Thapa, Janani Rajbhandari-Thapa, Saanie Sulley, Faustine Williams

**Affiliations:** 1 Division of Intramural Research, National Institute on Minority Health and Health Disparities, National Institutes of Health, 20852 Bethesda, MD USA; 2 Department of Epidemiology and Biostatistics, University of Georgia, Athens, GA USA; 3 Department of Health Policy and Management, University of Georgia, Athens, GA USA; 4 National Healthy Start Association, Washington, DC USA

**Keywords:** Mental health, Immigrants, First-generation, Foreign-born, Nativity, Disparities, Psychological, Biopsychosocial

## Abstract

**Background:**

Despite increasing studies on mental health among immigrants, there are limited studies using nationally representative samples to examine immigrants’ mental health and its potential biopsychosocial contributing factors, especially during the COVID-19 pandemic. We explored and estimated the influence of life satisfaction, social/emotional support, and other biopsychosocial factors on self-reported anxiety/depression symptoms among a nationally representative sample of first-generation immigrants in the U.S.

**Methods:**

We conducted a secondary data analysis using the 2021 National Health Interview Survey among first-generation adults aged ≥ 18 years (*n* = 4295). We applied survey weights and developed multivariable logistic regression model to evaluate the study objective.

**Results:**

The prevalence of daily, weekly, or monthly anxiety/depression symptoms was 10.22% in the first-generation immigrant population. There were 2.04% daily, 3.27% weekly, and 4.91% monthly anxiety/depression among the population: about 8.20%, 9.94%, and 9.60% experienced anxiety symptoms, whereas 2.49%, 3.54%, and 5.34% experienced depression symptoms daily, weekly, and monthly, respectively. The first-generation population aged 26–49 years were less likely to experience anxiety/depression daily, weekly, or monthly compared to those aged 18–25. Females (versus males) were more likely to experience anxiety/depression daily, weekly, or monthly. Those who identified as gay/lesbian had higher odds of experiencing anxiety/depression daily, weekly, or monthly compared to heterosexual persons. Relative to non-Hispanic White individuals, non-Hispanic Asian, Black/African American, and Hispanic individuals had lower odds, while other/multi-racial/ethnic groups were more likely to experience anxiety/depression daily, weekly, or monthly. A higher life satisfaction score was associated with lower odds of experiencing anxiety/depression daily, weekly, or monthly. Having social/emotional support sometimes/rarely or using healthcare within the past one/two years was associated with experiencing anxiety/depression daily, weekly, or monthly.

**Conclusions:**

The findings reveal significant burden of anxiety and depression among first-generation population in the U.S., with higher risks among subgroups like young adults, females, sexual minorities, and non-Hispanic White and other/multi-racial individuals. Additionally, individuals with lower life satisfaction scores, limited social/emotional support, or healthcare utilization in the past one or two years present increased risk. These findings highlight the need for personalized mental health screening and interventions for first-generation individuals in the U.S. based on their diversity and health-related risks.

## Background

First-generation immigrants (i.e., individuals who are foreign born or not born in their host country) often face higher socioeconomic challenges with associated stress than second, third, and higher generation individuals (native-born or born in the host country) [1–4]. First-generation immigrants in the United States (U.S.) in 2018 account for 13.7% of the U.S. population, which is close to the historic high in 1890 (14.8%) [5]. Labor force participation also increased from 17.2% in 2007 to 21.2% in 2017 for lawful immigrants [5]. Despite increasing participation in the labor force and important contribution to the U.S. economy, immigrants are largely understudied in mental health research. Immigrant-specific struggles, such as language barriers, social isolation, discrimination, and a loss of social support networks, can exacerbate existing mental health issues or trigger new ones among immigrants. The experience of acculturative stress, a psychological distress that results from the process of adapting to a new culture, may also contribute to mental health issues among immigrants [6]. Many immigrants might have experienced trauma, including war or persecution, in their country of origin, which can further complicate their mental health [7]. Post-migration stress and trauma have also been found to impact the mental health of these populations [8], suggesting the need to evaluate the potential risk factors for mental health conditions that can vary for immigrant subgroups, especially during pandemic like the COVID-19.

Mental health disorder symptoms, including anxiety and depression that are the most common symptoms, have increased risks for morbidity (e.g., cancer, cardiovascular diseases, stroke, diabetes, Alzheimer’s disease) and mortality [9–20]. Anxiety and depression symptoms, often assessed together to measure overall psychological distress or mental health, commonly co-occur with elevated disability severity [9, 15, 20–24]. The detrimental impacts of mental health disorder symptoms further highlight the need to assess these symptoms, especially anxiety and depression symptoms, among immigrant population who are generally understudied in the mental health field to augment the literature and enhance tailored mental health interventions.

The need for mental health research has become increasingly apparent during the COVID-19 pandemic. The pandemic has exacerbated existing mental health issues and also given rise to new ones, disproportionately impacting immigrant communities [25, 26]. Immigrants faced heightened health risks, economic challenges, and social isolation due to the pandemic [27, 28]. Immigrants may not only encounter unique challenges that lead to increasing prevalence of anxiety and depression, but also distinct barriers related to accessing mental health services including language, lack of insurance, and stigma surrounding mental health [29]. Lai and colleagues, for example, found that Chinese immigrants were less likely to seek help for mental health issues due to stigma and a lack of understanding about mental health [30]. Similarly, another study found that female Latino immigrants were less likely to use mental health services compared to non-immigrant Latinos due to financial and logistical barriers [31]. In recent years, there has been a growing interest in studying mental health and its associated factors among immigrants. In particular, one study examined the role of social support in the mental health of Somali refugees, revealed that high levels of social support were associated with better mental health outcomes [32]. Another study focused on the experiences of immigrant women in Canada, highlighting the importance of culturally sensitive care and the need to address social and structural determinants of health [33].

Despite increasing research on mental health among immigrants, there are limited studies using nationally representative samples to examine immigrants’ mental health, especially during the COVID-19 pandemic. An area less examined is life satisfaction, a significant determinant of overall wellbeing or quality of life, among immigrants and its association with mental health [34, 35]. The associated health benefits of life satisfaction include longer and healthier lives, better mental health, and reduced mortality risks [34–40]. Life satisfaction thus reduces the risk of experiencing mental health problems, including anxiety and depression [41–44]. Individuals with higher life satisfaction have lower mental health problems, particularly psychological distress, anxiety, depression, and suicidality [41–44]. Given the importance of life satisfaction, especially for immigrants who seek better socioeconomic conditions and safety in their host countries [39, 45], there is a need to evaluate the influence of life satisfaction on mental health among immigrants.

Given the distinctive acculturative stressors immigrants face, it is crucial to study mental health-related factors specific to the immigrant population to develop effective interventions and support systems for this population. Mental health is the central factor of biopsychosocial factors (i.e., interaction between biological, psychological, and socio-environmental experiences) among immigrants. That is, according to the biopsychosocial model or framework perspective, mental health conditions are easily influenced by biological (e.g., age, sex, weight), psychological (e.g., life satisfaction), and socio-environmental (e.g., income, education, sexual orientation, health utilization) factors among immigrants [46–57]. Understanding how biopsychosocial factors or experiences contribute to mental health issues among immigrants, during this COVID-19 pandemic, can inform the development of culturally sensitive and linguistically appropriate services during and after epidemiologic crisis. This can help ensure that immigrants receive the care they need to manage their mental health and lead healthy, productive lives in their new home countries and continue to make contributions to the U.S. economy. This study aimed to (a) explore the patterns/frequency of anxiety, depression, and anxiety/depression symptoms and (b) estimate the influence of life satisfaction, social support, and other biopsychosocial factors (i.e., sociodemographic characteristics, health utilization) on anxiety/depression symptoms among foreign-born or first-generation immigrant population during the COVID-19 pandemic. The insights obtained from this study will be timely and essential in informing the development of targeted and culturally sensitive interventions to mitigate the mental health impacts of the pandemic on immigrant populations.

## Methods

### Study design

We conducted a secondary data analysis using the 2021 National Health Interview Survey (2021 NHIS) deidentified public use file. NHIS is a nationally representative household-level cross-sectional survey conducted among the civilian noninstitutionalized population of the U.S. to assess health information and the demographic and socioeconomic characteristics of the population [35]. It is conducted annually among children (0–17 years) and adults ≥ 18 years by the National Center for Health Statistics (NCHS). The NHIS involves a stratified, multistage, complex clustered sampling of random dwelling units and participants [35]. First, the U.S. is partitioned into geographic areas including counties, a small group of contiguous counties, or a metropolitan area within state boundaries. Next, geographical areas are divided into strata based on population density (i.e., urban and rural counties) within some states (i.e., populous states), while all the geographical areas form one stratum within the remaining states. Third, clusters of addresses or houses are systematically defined within each stratum. Finally, a child and an adult are randomly chosen from each selected household to form the NHIS sample. The 2021 survey was conducted between January and December 2021. The data were collected through in-person and telephone interviews. The 2021 NHIS includes a total of 29,482 adults (response rate was 50.9%) [35]. For this analysis, we conducted a subpopulation analysis using the sample of foreign-born or first-generation adults, individuals not born in the U.S. or U.S. territory (*n* = 4709). We performed a complete case analysis resulting in 4295 first-generation adults.

### Measures

#### Self-reported measures anxiety/depression

The dependent variable was self-reported anxiety/depression symptoms. Anxiety symptoms were assessed by asking the participants to self-report, “How often do you feel worried, nervous, or anxious? Would you say daily, weekly, monthly, a few times a year, or never?” Depression symptoms were evaluated by asking the participants, “How often do you feel depressed? Would you say daily, weekly, monthly, a few times a year, or never?” The response options were the same for anxiety and depression symptoms: 1 = Daily, 2=, Weekly, 3 = Monthly, 4 = A few times a year, 5 = Never, 7 = Refused, 8 = Not Ascertained, 9 = Don’t Know. We combined anxiety and depression symptoms to form anxiety/depression symptoms. Similar to the literature [58], we dichotomized the anxiety/depression symptoms into a positive outcome if the participants experienced the symptoms of either anxiety or depression daily, weekly, or monthly; otherwise, the participants were assigned a negative outcome.

### Biopsychosocial factors

Life satisfaction was assessed via a single-item measure by asking the participants, “Using a scale of 0 to 10, where 0 means “very dissatisfied” and 10 means “very satisfied,” how do you feel about your life as a whole these days?” Higher values represent higher life satisfaction among the participants.

Social/emotional support frequency was measured through “How often do you get the social and emotional support you need? Would you say always, usually, sometimes, rarely, or never?” The response options included 1 = Always, 2 = Usually, 3 = Sometimes, 4 = Rarely, 5 = Never, 7 = Refused, 8 = Not Ascertained, or 9 = Don’t Know. We recoded this variable as always/usually, sometimes/rarely, or never.

We also analyzed the following independent variables: age (18–25, 26–34, 35–49, 50–64, 65 or older), biological sex (male or female), sexual orientation (heterosexual, lesbian/gay, bisexual, or other [something else, or uncertain]), citizenship status (citizen or non-citizen), race/ethnicity (non-Hispanic White, non-Hispanic Black/African American, non-Hispanic Asian, Hispanic, or other race/ethnic group [American Indian or Alaska Native, Native Hawaiian or other Pacific Islander, or other single and multiple races]), level of education completed (Less than high school, high school diploma or G.E.D., some college/associate degree, or college or higher degree), family income to poverty ratio (0.00–11+), health insurance status (Not insured or insured), marital status (Single/never married, married, or divorced/separated/widowed), acculturation/length of stay in the U.S. (Less than 5 years or ≥ 5 years), and BMI (Healthy weight [BMI = 18.5 to < 25], underweight [BMI < 18.5], Overweight [BMI ≥ 25 to < 30], or obese [BMI ≥ 30). Healthcare utilization was assessed by asking, “About how long has it been since you last saw a doctor or other health professional about your health?” Response options were 0 = Never, 1 = Within the past year (anytime less than 12 months ago), 2 = Within the last 2 years (1 year but less than 2 years ago), 3 = Within the last 3 years (2 years but less than 3 years ago), 4 = Within the last 5 years (3 years but less than 5 years ago), 5 = Within the last 10 years (5 years but less than 10 years ago), 6 = 10 years ago or more, 7 = Refused, 8 = Not Ascertained, or 9 = Don’t Know. We recategorized this variable as within the last three years or more/never used, within the last two years, or the past year or 12 months.

### Statistical analyses

**Fig. 1:**
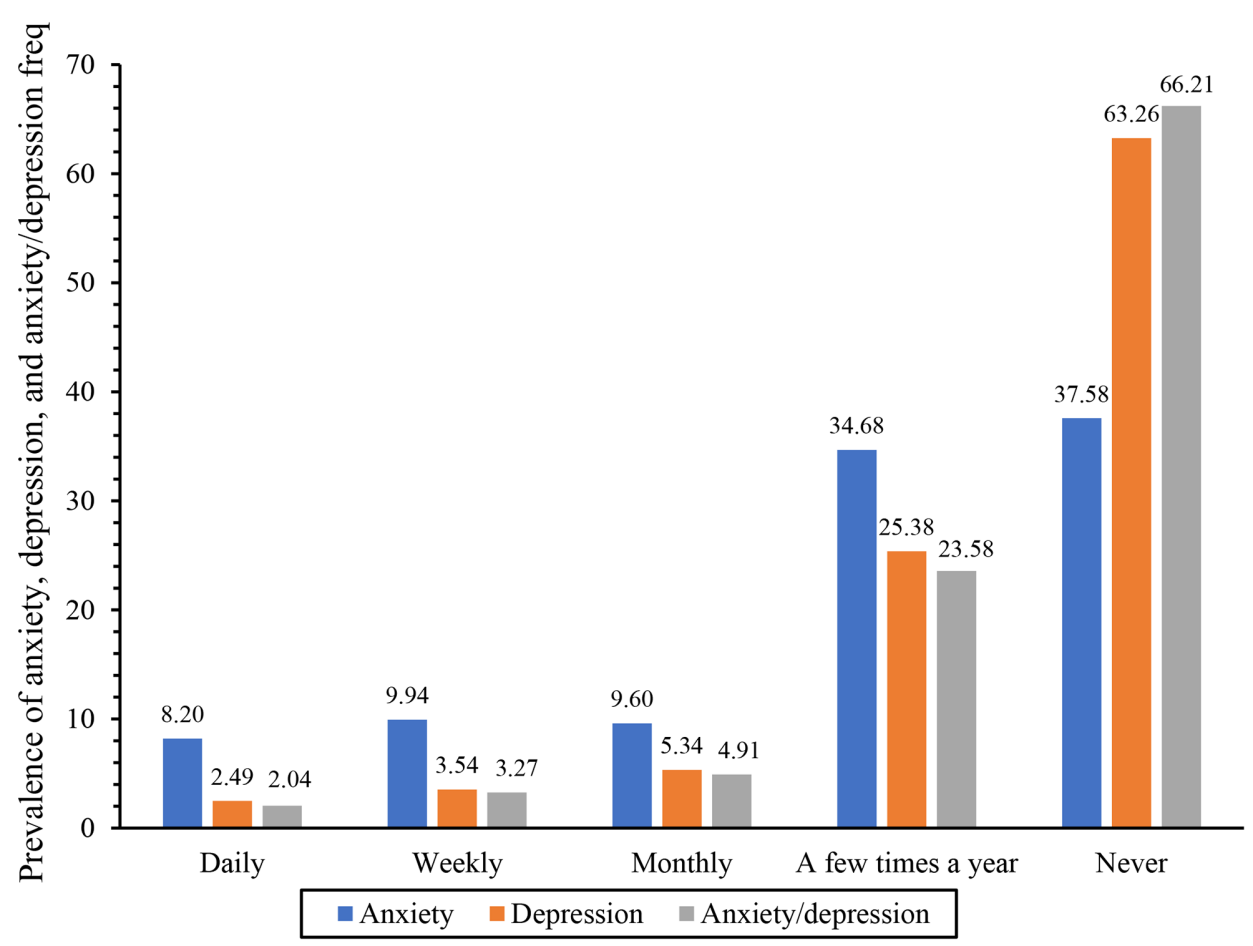
Prevalence of anxiety, depression, and anxiety/depression symptoms among the foreign-born/first-generation population

We first generated patterns/frequency of anxiety, depression, and anxiety/depression symptoms among the first-generation population (Fig. 1). Next, we computed descriptive and bivariate statistics of anxiety/depression symptoms by the biopsychosocial risk factors among the first-generation population (Table 1). The bivariate statistics were calculated using Rao–Scott *χ*^2^ tests and t-test or ANOVA to determine differences in the frequency of anxiety/depression symptoms by the biopsychosocial risk factors. We used the Rao–Scott *χ*^2^ because of its widely use with design-based approach and accounts for complex survey or sampling design [59–61]. A statistical significance level of ≤ 0.0005, instead of < 0.05, was used at the bivariate analysis level to determine group differences to reduce uncertainty in the significance of the groups.

**Table 1:** Descriptive and bivariate statistics of the frequency of anxiety/depression symptoms by life satisfaction, and other sociodemographic characteristics among the first-generation population (Unweight *n* = 4295 and Weighted *N* = 40,990,369)

		Frequency of anxiety/depression		
	Overall Sample	A few times a year or never	Daily/weekly/monthly	
	n (%)	n (%)	n (%)	P-value
**Overall**		3,841 (89.78)	454 (10.22)	
**Age groups**				0.0002
18–25	223 (7.87)	191 (86.10)	32 (13.90)	
26–34	615 (15.25)	563 (92.69)	52 (7.31)	
35–49	1416 (33.04)	1296 (92.32)	120 (7.68)	
50–64	1160 (26.75)	1015 (87.18)	145 (12.82)	
65 and older	881 (17.09)	776 (88.03)	105 (11.97)	
**Sex**				0.0062
Male	1926 (48.11)	1760 (91.32)	166 (8.68)	
Female	2369 (51.89)	2081 (88.36)	288 (11.65)	
**Sexual orientation**				0.0032
Heterosexual	4126 (96.09)	3705 (90.13)	421 (9.87)	
Gay/Lesbian	54 (1.07)	41 (79.28)	13 (20.72)	
Bisexual	30 (0.64)	21 (71.73)	9 (28.27)	
Other/uncertain	85 (2.20)	74 (84.78)	11 (15.22)	
**Citizenship status**				0.2918
Citizen	2661 (58.43)	2365 (89.27)	296 (10.73)	
Non-citizen	1634 (41.58)	1476 (90.50)	158 (9.50)	
**Race/ethnicity**				0.0002
Non-Hispanic White	915 (18.02)	789 (86.97)	126 (13.03)	
Non-Hispanic Black/African American	361 (10.07)	329 (91.23)	32 (8.77)	
Non-Hispanic Asian	1275 (25.76)	1178 (91.60)	97 (8.40)	
Hispanic	1679 (44.86)	1496 (90.05)	183 (9.95)	
Other/Multi-racial group	65 (1.30)	49 (72.27)	16 (27.73)	
**Level of education completed**				0.1076
Less than high school	765 (20.76)	683 (89.79)	82 (10.21)	
High school diploma or GED	895 (24.31)	802 (90.62)	93 (9.38)	
Some college/associate degree	844 (18.68)	740 (87.00)	104 (13.00)	
**Family income to poverty ratio** **(Mean: SD)**	4295 (3.65: 2.93)	3841 (3.67: 2.92)	454 (3.47: 2.99)	0.2404
**Health insurance status**				0.1843
Not insured	579 (17.04)	526 (91.55)	53 (8.45)	
Insured	3716 (82.96)	3315 (89.42)	401 (10.58)	
**Marital status**				0.0004
Single/never married	828 (20.35)	726 (88.62)	102 (11.38)	
Married	2586 (64.86)	2363 (91.20)	223 (8.80)	
Divorced/Separated/Widowed	881 (14.80)	752 (85.16)	129 (14.84)	
**Acculturation/length of stay in the U.S.**				0.3301
Less than five years	263 (6.72)	242 (91.91)	21 (8.09)	
Five years or more	4032 (93.28)	3599 (89.63)	433 (10.37)	
**Life satisfaction (Mean: SD)**	4295 (8.41: 1.73)	3841 (8.58: 1.54)	454 (6.86: 2.42)	< 0.0001
**Social/emotional support frequency**				0.0001
Never	367 (8.69)	337 (91.66)	30 (8.34)	
Sometimes or rarely	705 (15.55)	555 (80.55)	150 (19.45)	
Always or usually	3223 (75.76)	2949 (91.46)	274 (8.54)	
**Healthcare utilization**				0.0001
Within the last three years or more/never used	383 (9.65)	359 (95.58)	24 (4.42)	
Within the last two years	541 (12.81)	498 (92.07)	43 (7.93)	
Within the past year	3371 (77.54)	2984 (88.68)	387 (11.32)	
**BMI status**				0.0347
Healthy weight	1651 (35.69)	1490 (89.99)	161 (10.01)	
Underweight	75 (1.66)	60 (77.76)	15 (22.24)	
Overweight	1598 (38.16)	1437 (90.45)	161 (9.56)	
Obese	971 (24.49)	854 (89.25)	117 (10.75)	

SD = standard deviation

We conducted a series of unadjusted logistic regression models to assess the association between anxiety/depression symptoms and each of the biopsychosocial risk factors (Table 2). Next, we used an adjusted multivariable logistic regression model to examine the association between the anxiety/depression symptoms and the biopsychosocial risk factors (Table 2); for each variable, the remaining variables were controlled for in the model. All the analyses, including the logistic regression analyses, were weighted using the NHIS 2021 sampling weight to account for the complex survey or sampling design (i.e., cluster, strata, and sampling weight) and offset nonresponse and produce nationally representative estimates. STATA 17.0 was used to conduct the analyses. We conducted a complete case analysis. The logistic regression models estimated adjusted odds ratios (AORs) with 95% confidence intervals (CIs) and crude or unadjusted ORs.

**Table 2:** Multivariable logistic regression analysis of the effects of biopsychosocial factors on anxiety/depression symptoms (Daily/weekly/monthly versus a few times a year or never)

	Crude OR (95% CI)	Adjusted OR (95% CI)
**Age groups**		
18–25	Ref	Ref
26–34	0.49** (0.29, 0.82)	0.52* (0.30, 0.91)
35–49	0.52** (0.33, 0.81)	0.58* (0.34, 0.98)
50–64	0.91 (0.57, 1.45)	0.84 (0.49, 1.45)
65 and older	0.84 (0.52, 1.36)	0.70 (0.39, 1.26)
**Sex**		
Male	Ref	Ref
Female	1.39** (1.10, 1.75)	1.39* (1.05, 1.84)
**Sexual orientation**		
Heterosexual	Ref	Ref
Gay/Lesbian	2.39* (1.14, 5.01)	2.43* (1.02, 5.77)
Bisexual	3.60** (1.39, 9.32)	2.18 (0.70, 6.83)
Other/uncertain	1.64 (0.80, 3.37)	1.44 (0.63, 3.31)
**Citizenship status**		
Citizen	Ref	Ref
Non-citizen	0.87 (0.68, 1.12)	0.97 (0.71, 1.34)
**Race/ethnicity**		
Non-Hispanic White	Ref	Ref
Non-Hispanic Black/African American	0.64* (0.41, 0.99)	0.62 (0.38, 1.02)
Non-Hispanic Asian	0.61** (0.44, 0.85)	0.60** (0.42, 0.86)
Hispanic	0.74* (0.55, 0.99)	0.83 (0.57, 1.20)
Other/Multi-racial group	2.56** (1.28, 5.10)	2.64* (1.08, 6.42)
**Level of education completed**		
Less than high school	Ref	Ref
High school diploma or GED	0.91 (0.63, 1.32)	0.95 (0.62, 1.45)
Some college/associate degree	1.31 (0.90, 1.91)	1.13 (0.72, 1.77)
**Family income to poverty ratio**	0.98 (0.94, 1.02)	1.00 (0.95, 1.05)
**Health insurance status**		
Not insured	Ref	Ref
Insured	1.28 (0.89, 1.85)	0.79 (0.50, 1.24)
**Marital status**		
Single/never married	Ref	Ref
Married	0.75 (0.56, 1.01)	0.95 (0.67, 1.36)
Divorced/Separated/Widowed	1.36 (0.95, 1.94)	1.20 (0.79, 1.82)
**Acculturation/length of stay in the U.S.**		
Less than five years	Ref	Ref
Five years or more	1.32 (0.76, 2.29)	1.20 (0.65, 2.23)
**Life satisfaction**	0.65*** (0.61, 0.70)	0.67*** (0.63, 0.72)
**Social/emotional support frequency**		
Never	Ref	Ref
Sometimes or rarely	2.65*** (1.60, 4.40)	2.39** (1.42, 4.04)
Always or usually	1.03 (0.62, 1.69)	1.18 (0.70, 1.98)
**Healthcare utilization**		
Within the last three years or more/never used	Ref	Ref
Within the last two years	1.86 (0.99, 3.52)	2.05* (1.03, 4.07)
Within the past year	2.76*** (1.64, 4.67)	2.51** (1.39, 4.55)
**BMI status**		
Healthy weight	Ref	Ref
Underweight	2.57* (1.23, 5.38)	1.92 (0.89, 4.18)
Overweight	0.95 (0.73, 1.24)	1.04 (0.75, 1.42)
Obese	1.08 (0.81, 1.45)	1.10 (0.79, 1.55)

*p < 0.05. **p < 0.01. ***p < 0.001

## Results

### Sociodemographic characteristics of the first-generation population

Table 1 presents the descriptive and bivariate statistics. Most of the first-generation population was 35–49 years of age (33.04%), female (51.89%), heterosexual (96.09%), U.S. citizen (58.43%), Hispanic (44.86%), completed college or higher education (36.26%), mean family income to poverty ratio of 3.65 (SD = 2.93), had insurance (82.96%), married (64.86%), lived in the U.S. for five years or more (93.28%), had a mean life satisfaction score of 8.41 (SD = 1.73), had social/emotional always or usually (75.76%), used healthcare within the past 12 months (77.54%), or were overweight (38.16%).

### Prevalence of anxiety/depression, anxiety, and depression

The prevalence of daily, weekly, or monthly anxiety/depression symptoms was 10.22% in the population (Table 1). There were 2.04% daily, 3.27% weekly, and 4.91% monthly anxiety/depression symptoms among the population (Fig. 1); about 8.20%, 9.94%, and 9.60% experienced anxiety symptoms, whereas 2.49%, 3.54%, and 5.34% experienced depression symptoms daily, weekly, and monthly, respectively. Overall, anxiety symptoms were more frequent compared to depression symptoms and anxiety/depression symptoms. We observed statistically significant differences between anxiety and depression patterns (*p* < 0.0001).

There were significant differences in the frequency of anxiety/depression symptoms based on the biopsychosocial risk factors (Table 1). Of the population with daily, weekly, or monthly anxiety/depression symptoms, the majority were 18–25 years (13.90%), females (11.65%), bisexual individuals (28.27%), non-Hispanic White (13.03%) or other racial/ethnic groups (27.73%), were divorced/separated/widowed (14.84%), had a lower life satisfaction score (mean: 6.86, SD = 2.42), sometimes or rarely had social/emotion support (19.45%), used healthcare within the past 12 months (11.32%), and were underweight (22.24%).

### Biopsychosocial factors associated with anxiety/depression

The odds ratio estimates are presented in Table 2. The first-generation population aged 26–34 (AOR: 0.52; 95% CI: 0.30, 0.91) or 35–49 (AOR: 0.58; 95% CI: 0.34, 0.98) years were less likely to experience anxiety/depression daily, weekly, or monthly compared to those aged 18–25 years. Females, compared to males, were more likely to experience anxiety/depression daily, weekly, or monthly (AOR.: 1.39; 95% CI: 1.05, 1.84). Those who identified as gay/lesbian individuals had higher odds of experiencing anxiety/depression daily, weekly, or monthly (AOR: 2.43; 95% CI: 1.02, 5.77) compared to their heterosexual counterparts. Non-Hispanic Asian (AOR: 0.60; 95% CI: 0.42, 0.86), Black/African American (Crude OR: 0.64; 95% CI: 0.41, 0.99 and AOR: 0.62; 95% CI: 0.38, 1.02), and Hispanic (Crude OR: 0.74; 95% CI: 0.55, 0.99 and AOR: 0.83; 95% CI: 0.57, 1.20) individuals had lower odds of experiencing anxiety/depression daily, weekly, or monthly compared to non-Hispanic White individuals. The AORs were not statistically significant for Black/African American and Hispanics individuals though. The odds were higher, however, for other racial/ethnic groups (AOR: 2.64; 95% CI: 1.08, 6.42). A higher life satisfaction score was significantly associated with lower odds of experiencing anxiety/depression daily, weekly, or monthly (AOR: 0.67; 95% CI: 0.63, 0.72). Having social/emotional support sometimes or rarely (AOR: 2.39; 95% CI: 1.42, 4.04) or using healthcare within the past 12 months (AOR: 2.51; 95% CI: 1.39, 4.55) and the past two years (AOR: 2.05; 95% CI: 1.03, 4.07) was significantly associated with experiencing anxiety/depression daily, weekly, or monthly (Table 2).

## Discussion

This study contributes to the immigrant mental health literature, by exploring anxiety/depression symptoms based on perceived social/emotional support, life satisfaction, and biopsychosocial risk factors among first-generation populations in the U.S. during the COVID-19 pandemic. This is particularly important given the often underrepresentation of these subpopulations in health research [62, 63]. As shown in our finding, the differences in the prevalence of anxiety and depression offer substantial evidence that although these disorder symptoms often co-occur, they still maintain distinct patterns of expression, as observed by Kessler et al. [64]. Contrary to previous findings, which suggested a ‘happiness advantage’ and a ‘healthy immigrant effect’ with better behavioral, physical, and mental health outcomes among immigrants, especially first-generation individuals and those with lower level of acculturation or years since immigration [49, 65–67], our study indicates a more nuanced and complex reality. Our findings revealed that anxiety/depression varied within the first-generation population, indicating that the ‘healthy immigrant effect’ paradox may not be applicable to all immigrant populations due to the diversity in the immigrant population. For instance, we found that individuals who reported receiving social/emotional support “sometimes” or “rarely” had higher odds of experiencing anxiety/depression symptoms, suggesting a potential protective role of stable social support against mental health issues [68–70]. These findings align with existing literature highlighting the positive influence of life satisfaction and social/emotional support on mental health outcomes [68, 71, 72].

The variation in the prevalence of depression/anxiety among the first-generation population observed in this study suggests that some groups may be more susceptible to certain mental health conditions than others. For example, we found that younger age groups (18–25 years) had higher odds of experiencing anxiety/depression, aligning with previous literature indicating that younger individuals often face unique mental health stressors [73, 74]. These findings may be associated with diverse social factors such as acculturation, establishing independence, beginning careers, and forming relationships, which may contribute to an increased risk of mental health issues [75, 76].

Females had a higher likelihood of experiencing anxiety/depression symptoms, and this finding may be explained by hormonal differences, differential stress responses, and social factors contributing to this gender disparity in mental health [77–79]. Another potential explanation for this finding could be the compounded effect of gender discrimination and migration status [80]. Our results further show that the frequency of healthcare utilization was significantly associated with the reported symptoms of depression and anxiety. Individuals who had utilized healthcare services within the past year reported a higher frequency of these symptoms than those who had used these services less frequently or never. These results support previous findings in the general U.S. population, which indicate that individuals with mental health issues, such as depression and anxiety, are more likely to utilize healthcare services [81, 82]. This observation could be due to the need for medical treatment and follow-up for these conditions, or healthcare use for health needs other than mental health needs. Despite the high burdens of mental health problems among immigrants, they are generally less likely to use mental health services due to stigma, language barriers, lack of insurance and documentation, and turning to family, friends, or religious leaders for care [48, 83]. The possibility of unmet mental health needs or barriers to effective mental health care should be explored in future studies to expand understanding of the heterogeneity of the link between immigrant mental health and healthcare utilization over time.

Further, young adults, females, sexual minorities, and those from other/multi-racial groups displayed a higher prevalence of anxiety and depression symptoms. The minority stress theory by Meyer could aid in further explaining this phenomenon [84]. This theory suggests that individuals with minority status (e.g., racial/ethnic, sexual identity) experience unique forms of stress related to their marginalized positions in society, leading to elevated rates of mental health disorders [84]. Self-identified gay and lesbian individuals, who already face sexual orientation-related stressors, experience additional stress as a part of the immigrant subpopulation. More than one in five respondents who self-reported as gay/lesbian or bisexual reported daily/weekly or monthly anxiety and depression symptoms, and about 28% of the other or multi-racial group reported a high frequency of these symptoms. Participants self-identifying as belonging to a sexual minority subgroup and other/multi-racial groups were significantly more likely to experience daily/weekly or monthly anxiety and depression symptoms. First-generation individuals that identify as gay/lesbian showed a higher likelihood of experiencing anxiety/depression symptoms, which is consistent with the findings of other studies demonstrating that sexual minorities often experience unique social stressors, such as discrimination and social stigma, contributing to poorer mental health outcomes [85, 86]. Other studies have also established the relationship between the intersectional nature of multiple minority status and health outcomes [87–89]. These show the need for interventions that account for the cultural and contextual experiences of immigrants as a whole and address the unique stressors these groups face.

Our findings further suggest differences in the frequency of self-reported depression and anxiety symptoms by race/ethnicity among the first-generation immigrant population. This is consistent with other studies that found persistent mental health disparities by race and ethnicity among the general U.S. population [90, 91]. Among all racial/ethnic groups, individuals identified as other/multi-racial (i.e., American Indian or Alaska Native, Native Hawaiian or other Pacific Islander, or other single and multiple races) had the highest prevalence and odds of experiencing anxiety/depression daily, weekly, or monthly, significantly exceeding that of any other group. This finding implies the existence of unique mental health stressors within this population [92, 93]. Complex and multifaceted minority stressors (e.g., prejudice, discrimination, racism) might have exacerbated the mental health of other/multi-racial immigrant groups [87, 88, 92, 93]. Diverse factors, such as group-specific discrimination and subsequent mental health gap, have been reported [94]. The higher levels of anxiety and depression observed among first-generation immigrants of other-multi-racial backgrounds might also be a result of a perceived lack of belonging and solidarity. The potential conflict between their physical identity and self-identity could create stress and increase the likelihood of experiencing anxiety and depression [87, 88, 92, 93]. Several studies found that perceived discrimination (e.g., anti-immigrant and refugee discrimination) can negatively affect the health of immigrant and refugee populations, including their mental health [95, 96].

The evidence in the literature on Hispanic health paradox suggests that Hispanic immigrants have better health outcomes compared to native populations and other populations, even when they experience discrimination and other social determinants of health disparities [97]. Similarly, we observed racial differences in anxiety/depression between non-Hispanic White and Hispanic populations within the first-generation populations. Hispanic individuals were less likely to experience anxiety/depression daily/weekly/monthly. These findings underscore the need for tailored mental health interventions that account for the unique experiences and stressors these at-risk immigrant subgroups face. They also highlight the need for further research to delineate disparities within immigrant populations more clearly to ensure that mental health interventions are accurately tailored for the most at-risk subgroups.

This study has some limitations. Firstly, it is a cross-sectional study and therefore the findings are limited to association instead of causal relationships. The self-reported nature of depression and anxiety is subject to an individual’s self-perception bias, and willingness to disclose such information may impact the accuracy of mental health status among the population of interest. The willingness to disclose mental health symptoms may be particularly relevant to immigrant populations because of the stigma often associated within these communities [83]. The measurement and categorization of the frequency of anxiety/depression symptoms in this study did not assess the degree (i.e., normal, mild, moderate, and severe) of anxiety/depression symptoms. Objective assessment of the symptoms might lead to more accurate information about these mental health disorder symptoms. The diversity of the U.S. immigrant population necessitates a more detailed subgroup analysis to better understand the unique factor impacting the substantial U.S. immigrant population. Future studies using longitudinal data could aid in establishing causality and accounting for the cultural contexts of the immigrant population.

## Conclusions

Our study underlines the significant burden of anxiety and depression among the first-generation population in the U.S., with higher prevalence and risks observed among specific subgroups like young adults, females, sexual minorities, and other/multi-racial individuals. The study also highlights the significant associations between these mental health outcomes and biopsychosocial factors such as life satisfaction, social/emotional support, and healthcare utilization. These findings further shed light on the need for personalized mental health screening and interventions for first-generation individuals in the U.S. considering the diversity of immigrant populations and their health-related risks. Also, it reveals the importance of strategies to address biopsychosocial determinants and mental health needs facing immigrants in the U.S. Furthermore, considering the heterogeneity and rapidly growing immigrant populations, research is recommended to explore specific at-risk subgroups, by examining potential barriers to effective mental health care, and implementing longitudinal and intervention studies to improve mental health outcomes.

## Data Availability

The datasets generated by the survey research during and/or analyzed during the current study are available in the CDC database repository, https://www.cdc.gov/nchs/nhis/2021nhis.htm.
